# Protective effects of *Helicobacter pylori* membrane vesicles against stress and antimicrobial agents

**DOI:** 10.1099/mic.0.000934

**Published:** 2020-05-28

**Authors:** Benjamin Oliver Murray, Robin Andrew Dawson, Lolwah Mohammad Alsharaf, Jody Anne Winter

**Affiliations:** ^1^​ School of Science and Technology, Nottingham Trent University, Nottingham, NG11 8NS, UK; ^2^​ Centre for Urological Biology, Department of Renal Medicine, Division of Medicine, University College London, London, UK; ^3^​ School of Environmental Sciences, University of East Anglia, Norwich Research Park, Norwich, NR4 7TJ, UK; ^4^​ Al-Amiri Hospital, Ministry of Health, Kuwait City, Kuwait

**Keywords:** antimicrobial, antibiotic resistance, *Helicobacter pylori*, membrane vesicles, survival, stress

## Abstract

Outer-membrane vesicles (OMVs) produced by *Helicobacter pylori* deliver bacterial components to host cells, provide a mechanism for stabilization of secreted components and may allow the bacteria to exert ‘long-range’ effects in the gastric niche, promoting persistence. In addition to their well-characterized host cell interactions, membrane vesicles improve stress survival in other bacterial species, and are constitutively produced by both pathogenic and non-pathogenic bacteria. We aimed to determine whether OMVs could improve *H. pylori* survival of a range of stressors. The effects of purified OMVs on the resistance of *H. pylori* to a range of environmental and antimicrobial stresses were determined using growth curves and survival assays. Addition of purified OMVs to *H. pylori* cultures provided dose-dependent protection against hydrogen peroxide-mediated killing. Supplementation with OMVs also partially protected *H. pylori* against the bactericidal effects of the antibiotics clarithromycin and levofloxacin, but not against amoxicillin nor metronidazole. Addition of purified OMVs allowed *H. pylori* to grow in the presence of inhibitory concentrations of the antimicrobial peptide LL-37. In the presence of 50 µg OMVs ml^−1^, significantly enhanced *H. pylori* growth was observed at higher LL-37 concentrations compared with lower LL-37 concentrations, suggesting that OMV–LL-37 interactions might facilitate release of growth-promoting nutrients. Taken together, these data indicate that production of membrane vesicles could help *H. pylori* to survive exposure to antibiotics and host antimicrobial defences during infection.

## Introduction

*Helicobacter pylori* is a Gram-negative, microaerophilic bacterium that infects the human stomach during early childhood. If untreated, infection persists lifelong despite a robust immune response [1] and causes asymptomatic gastritis, which may progress to ulceration and the development of gastric cancer [2, 3]. Treatment of *H. pylori* infection typically comprises a combination of amoxicillin with either clarithromycin or metronidazole administered with a proton pump inhibitor, but the failure rates of first-line triple therapies, particularly those containing clarithromycin [4], have been climbing. A range of alternative triple and quadruple therapies are now recommended, depending on the local antibiotic-resistance rates [5]. Levels of antibiotic resistance in *H. pylori* are escalating [4–7] and clarithromycin-resistant *H. pylori* was recently listed as one of the world’s highest priority antibiotic-resistant pathogens of concern by the World Health Organization [8]. There is a need for alternative therapies and better understanding of how *H. pylori* is able to persist lifelong in the harsh gastric environment.

Gram-negative bacteria, including *H. pylori *, constitutively release outer-membrane vesicles (OMVs) during normal growth [9, 10]. OMVs are small (20–300 nm), spherical vesicles predominantly containing outer membrane and periplasmic components from the bacterial cell [11]. *H. pylori* OMVs contain virulence factors including the toxin VacA [10, 12, 13] and are readily taken up by host cells [14, 15].

Roles for bacterial OMVs in host–pathogen interactions have been widely reported (reviewed by Schwechheimer and Kuehn, and MacDonald and Kuehn [11, 16]), but non-pathogenic bacteria also produce OMVs. Production of OMVs is upregulated in response to, and associated with survival of, stress in *Pseudomonas aeruginosa* [17] and *Escherichia coli* [18, 19], and OMVs contribute to bacterial survival of antibiotic treatment in *E. coli* [20, 21] and *Pseudomonas syringae* [22].

Protective effects of OMVs against oxidative stress in *H. pylori* were recently reported using strains P12 and 18943 [23]. If the production of OMV can help *H. pylori* to survive stressors such as the host immune response and antibiotic treatment, then it might be possible to increase the susceptibility of *H. pylori* to immunity and therapy, and/or reduce the virulence of the infection, by designing new therapies that interfere with vesiculation.

In this study, we aimed to determine whether OMVs could protect *H. pylori* against a range of stressors. Hydrogen peroxide and the cathelicidin derivative LL-37, a cationic antimicrobial peptide involved in the human gastric mucosal defence against *H. pylori* [24], were used to simulate immune-mediated stressors, and the protective effects of OMVs against antibiotics commonly used to treat *H. pylori* infections were also assessed.

## Methods

### Culture of *H. pylori*

*H. pylori* strain 60190 was provided by Professor John Atherton and his team at the University of Nottingham, UK. The bacteria were routinely cultured on blood agar base no. 2 (Oxoid) supplemented with 7.5 % defibrinated horse blood (TCS Biosciences) under microaerobic conditions (85 % N_2_, 10 % CO_2_, 5 % O_2_) at 37 °C.

### Purification of OMVs

For most of the reported assays, OMVs were isolated from late exponential or early stationary phase broth cultures of *H. pylori* in serum-free media [brain heart infusion (BHI) broth supplemented with 0.2 % β-cyclodextrin]. For some of the antimicrobial survival assays, to improve the yield of OMVs (since *H. pylori* growth in serum-free liquid media is very slow), OMVs were isolated directly from bacteria that were grown on agar plates then re-suspended in media. Bacterial cells were removed by centrifugation at 4000 ***g*** for 10 min and sequential filtration of the culture supernatant through 0.45 and 0.20 µm syringe filters. OMVs were purified from the culture supernatants by centrifugation at 100 000 ***g*** for 2 h at 4 °C, with a preceding 40 % ammonium sulfate precipitation step to concentrate the secreted proteins and OMVs from larger culture volumes, as previously described [25]. The OMV pellets were washed using particle-free Dulbecco’s PBS (Sigma Aldrich) and finally re-suspended in 200–500 µl PBS. OMVs were quantified using a Pierce BCA protein assay (Fisher Scientific) and stored promptly at −20 °C until use.

### Hydrogen peroxide survival assay

*H. pylori* were grown for 24 h on blood agar and then suspended to OD_600_ 0.1 in Iso-Sensitest broth (Oxoid) + 5 % (v/v) FCS (Sigma Aldrich). Bacteria were mixed with a final concentration of 0–50 µg OMVs ml^−1^ or 0.1 % (w/v) bovine catalase in triplicate in sterile 96-well plates and then H_2_O_2_ was added to all wells to a final concentration of 1 mM. After 2.5 h incubation at 37 °C, samples were taken from each well and diluted into 1 % (w/v) bovine catalase to inactivate any residual H_2_O_2_ before Miles and Misra determination of the number of c.f.u. ml^−1^.

### Growth inhibition assay

*H. pylori* were grown for 24 h on blood agar and then suspended in BHI broth + 0.2 % β-cyclodextrin to OD_600_ 0.1, supplemented with either 50 µg OMVs ml^−1^ in PBS or an equal volume of PBS without OMVs. Bacteria with and without OMVs were then incubated with 0.25–1.0 µg amoxicillin ml^−1^ (Sigma Aldrich) or 1.25–5.0 µg LL-37 ml^−1^ (InvivoGen) in 96-well plates under microaerobic conditions, and bacterial growth was monitored by measuring the OD_600_ at 24 h intervals for 1 week.

### Antimicrobial survival assays

Bacteria were adjusted to OD_600_ 0.1 in BHI broth + 0.2 % β-cyclodextrin and treated with the antimicrobial peptide LL-37 (InvivoGen) or the antibiotics amoxicillin, clarithromycin, metronidazole or levofloxacin (all from Sigma Aldrich) at the concentrations indicated in the figures, in the presence of 0–25 µg purified OMVs ml^−1^. Survival assays were set up in triplicate wells in sterile 96-well plates, in a total volume of 100 µl per well. After incubation in microaerobic conditions at 37 °C for 3 h, the surviving bacteria were quantified by serial dilution and plating out. Data were expressed as c.f.u. ml^−1^ and as percentage survival compared to the bacterial c.f.u. ml^−1^ in untreated control wells.

### Heat treatment of OMVs

Heat-treated OMVs were prepared by heating purified OMVs at 80 °C for 10 min, cooling to room temperature and then briefly centrifuging the heated OMV suspension to bring all liquid to the bottom of the tube.

### Statistical analysis

GraphPad Prism 8.1.2 was used for statistical analysis and figure generation. The tests used are indicated in each figure.

## Results

### Membrane vesicles can protect *H. pylori* against hydrogen peroxide-mediated killing in a dose-dependent manner

**Fig. 1.**
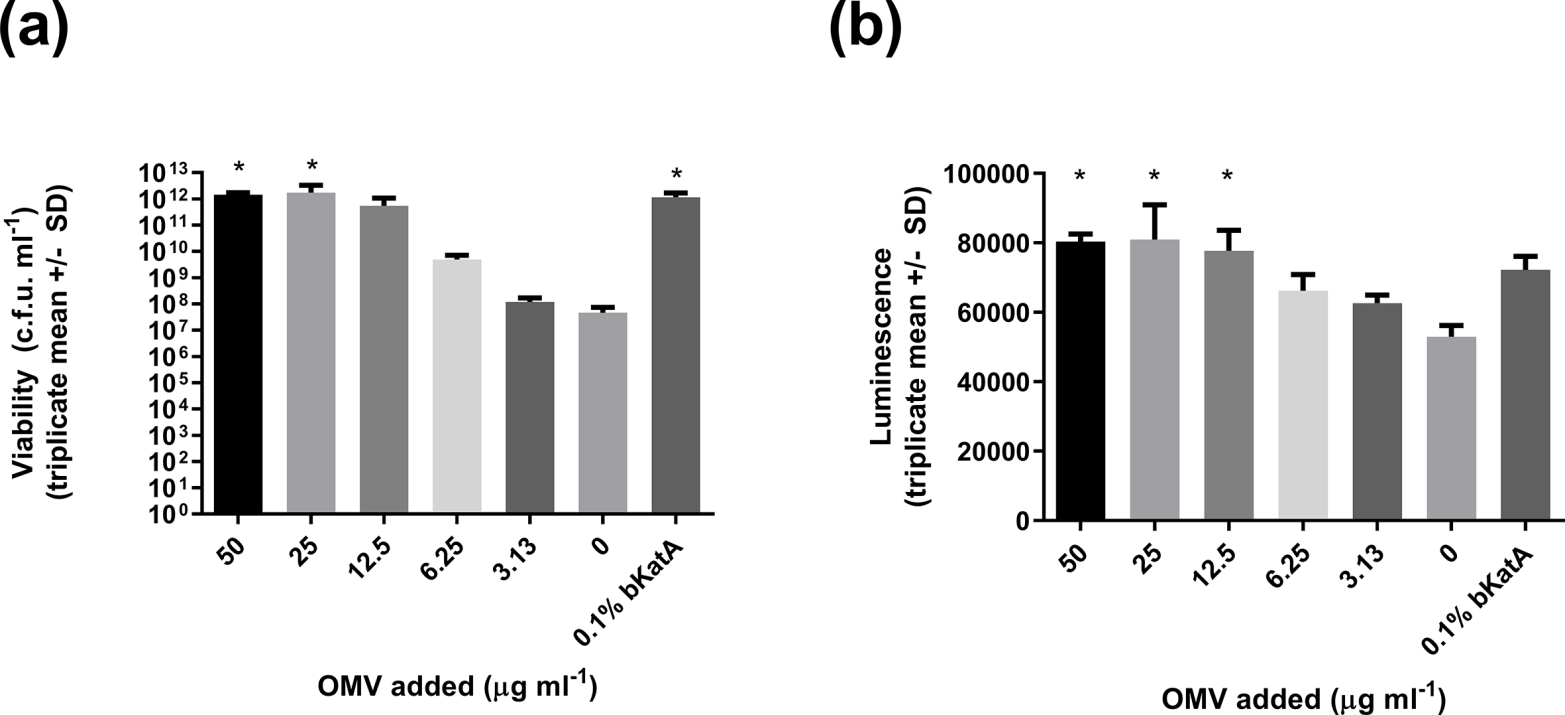
Viability of *H. pylori* in the presence of 1 mM H_2_O_2_ is restored by supplementation with OMVs. Bacteria were incubated at 37 °C in Iso-Sensitest broth, 5 % (v/v) FCS with 1 mM H_2_O_2_ and viability determined after 2.5 h by the Miles and Misra method. Triplicate means ± sd are shown. Bacterial viability was reduced by H_2_O_2_ treatment compared to the control in which H_2_O_2_ was inactivated using 0.1 % (w/v) bovine catalase (bKatA). Bacterial viability was restored to control levels by the addition of purified OMVs, in a dose-dependent manner. Asterisks indicate statistically significant differences compared to H_2_O_2_-treated bacteria without the addition of bKatA or OMVs (non-parametric Kruskal–Wallis test with multiple comparisons by Dunn’s test; multiplicity corrected *P* values reported; *P<0.05).

*H. pylori* OMVs are enriched with catalase [23], which is thought to contribute to bacterial survival of oxidative stress in the human stomach. We treated *H. pylori* with 1 mM H_2_O_2_ and measured bacterial survival using colony counts after 2.5 h. H_2_O_2_ treatment caused greater than 8-log reduction in c.f.u. ml^−1^ compared to a control that included 0.1 % bovine catalase to inactivate the H_2_O_2_ (Fig. 1).

To assess potential protective effects of OMVs against oxidative stress, OMVs were purified from stationary phase serum-free *H. pylori* culture supernatant by filtration and ultracentrifugation, suspended in PBS and confirmed free of viable bacteria by incubating samples on blood agar plates under microaerobic conditions for several days. Addition of OMVs protected *H. pylori* against H_2_O_2_-mediated killing in a dose-dependent manner (Fig. 1; *P*<0.05). Concentrations of OMVs at or above 12.5 µg ml^−1^ provided similar levels of protection against H_2_O_2_ to 0.1 % bovine catalase.

### Supplementation with membrane vesicles allows *H. pylori* to grow in the presence of the antimicrobial peptide LL-37

**Fig. 2.**
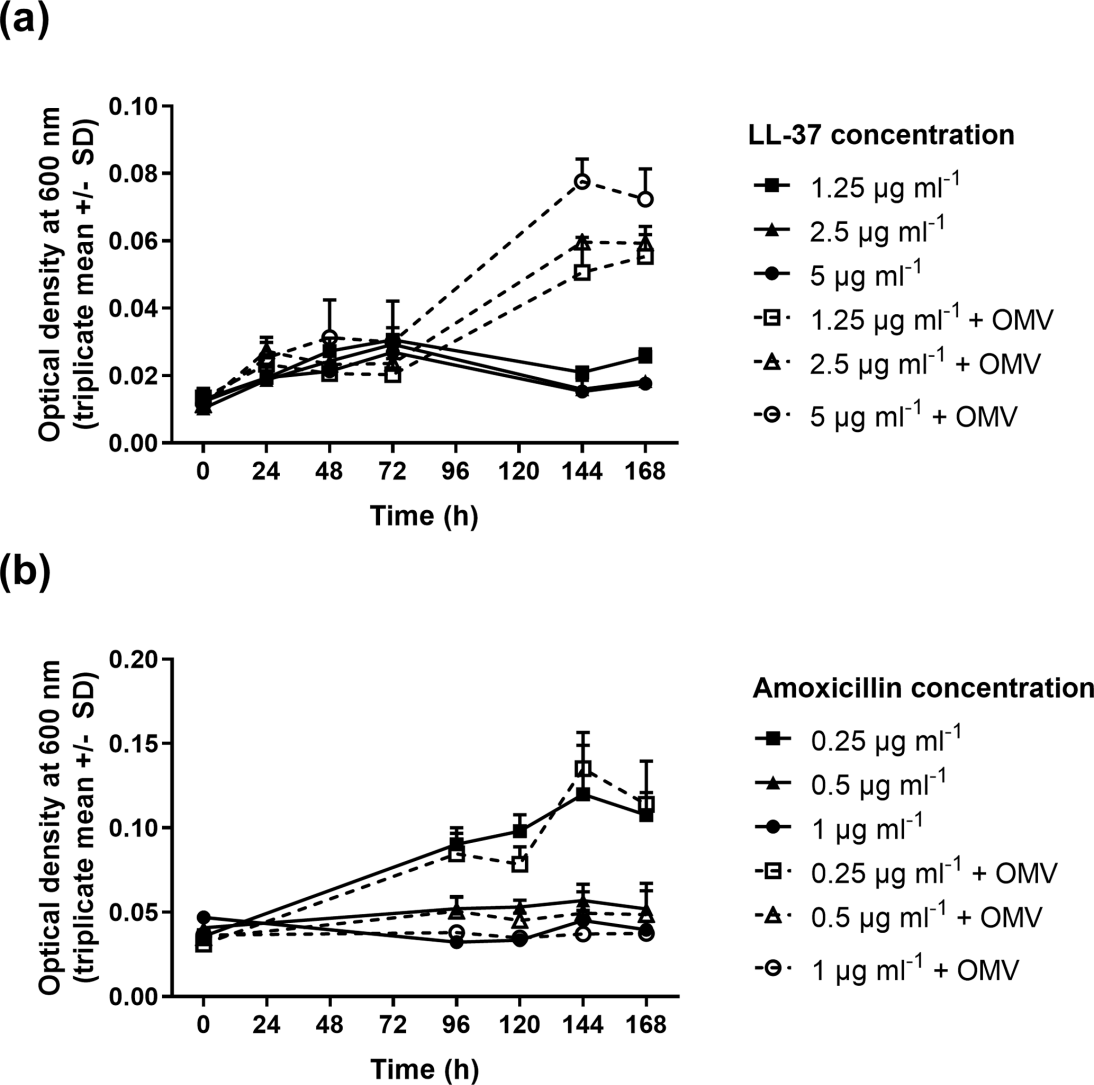
OMVs can protect *H. pylori* against the antimicrobial peptide LL-37, but not against amoxicillin. Bacteria were grown in different concentrations of LL-37 (a) or amoxicillin (b) as indicated by the keys, with (dashed lines) or without (solid lines) supplementation with 50 µg purified OMVs ml^−1^ . Growth of *H. pylori* 60190 in BHI + 0.2 % β-cyclodextrin was inhibited by 1.25–5 µg LL-37 ml^−1^ . When the bacteria were supplemented with 50 µg purified OMVs ml^−1^, growth was significantly enhanced (endpoint *P*<0.05, two-way ANOVA with Tukey post hoc tests) (a). OMVs did not enhance the growth of *H. pylori* in the presence of amoxicillin (b).

LL-37 is a cationic antimicrobial peptide involved in mucosal immune defence, and OMVs have previously been shown to protect *Vibrio cholerae* against this peptide [26]. LL-37 inhibited the growth of *H. pylori* when included in the growth media at concentrations between 1.25 and 5 µg ml^−1^, but growth in the presence of all concentrations of LL-37 was significantly enhanced (*P*<0.05) when the cultures were supplemented with 50 µg OMVs ml^−1^ (Fig. 2a). OMV-mediated growth promotion was greatest at the highest LL-37 concentration. OMVs were not able to enhance the growth of *H. pylori* in the presence of amoxicillin (Fig. 2b).

### Membrane vesicles can promote *H. pylori* survival of antibiotic treatment

**Fig. 3.**
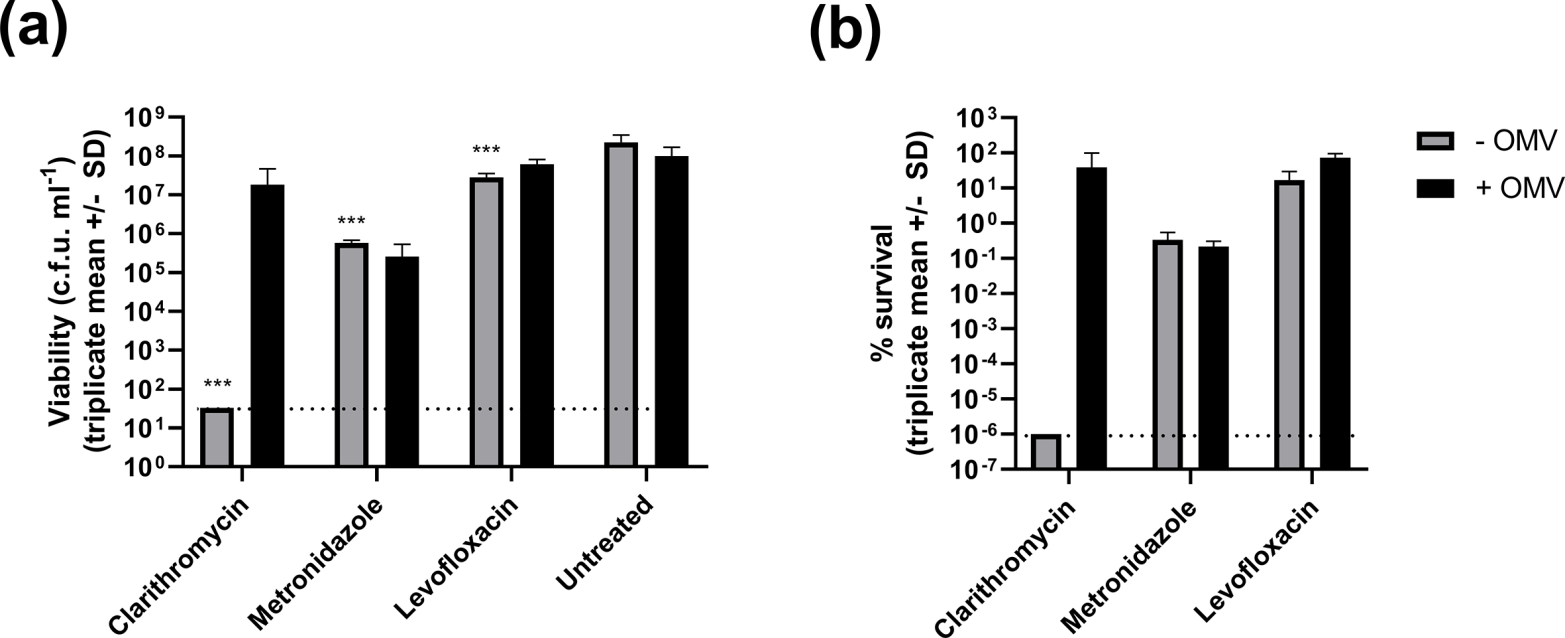
Protective effects of OMVs against clarithromycin treatment. *H. pylori* were incubated for 3 h in the presence of 10 µg clarithromycin ml^−1^, 100 µg metronidazole ml^−1^ or 10 µg levofloxacin ml^−1^. These concentrations of drug were sufficient to significantly reduce bacterial survival (***P<0.001, two-way ANOVA with Dunnett’s multiple comparisons tests versus the untreated control group). The limit of detection, 33 c.f.u. ml^−1^, is indicated by dashed lines. Supplementation with 25 µg purified OMVs ml^−1^ improved bacterial survival of clarithromycin and levofloxacin treatment, but not metronidazole. After OMV supplementation, bacterial survival was not significantly different to the untreated control group. Data shown are mean c.f.u. ml^−1^ ± sd (a) and percentage survival compared to the untreated control group (b) for three independent replicates.

*H. pylori* incubated for 3 h in the presence of clarithromycin, metronidazole or levofloxacin had significantly reduced survival compared to untreated control bacteria (*P*<0.001 for each antibiotic, two-way ANOVA with Dunnett’s multiple comparisons tests). Addition of 25 µg purified OMVs ml^−1^ improved bacterial survival in the presence of clarithromycin and levofloxacin, but not metronidazole (Fig. 3).

**Fig. 4.**
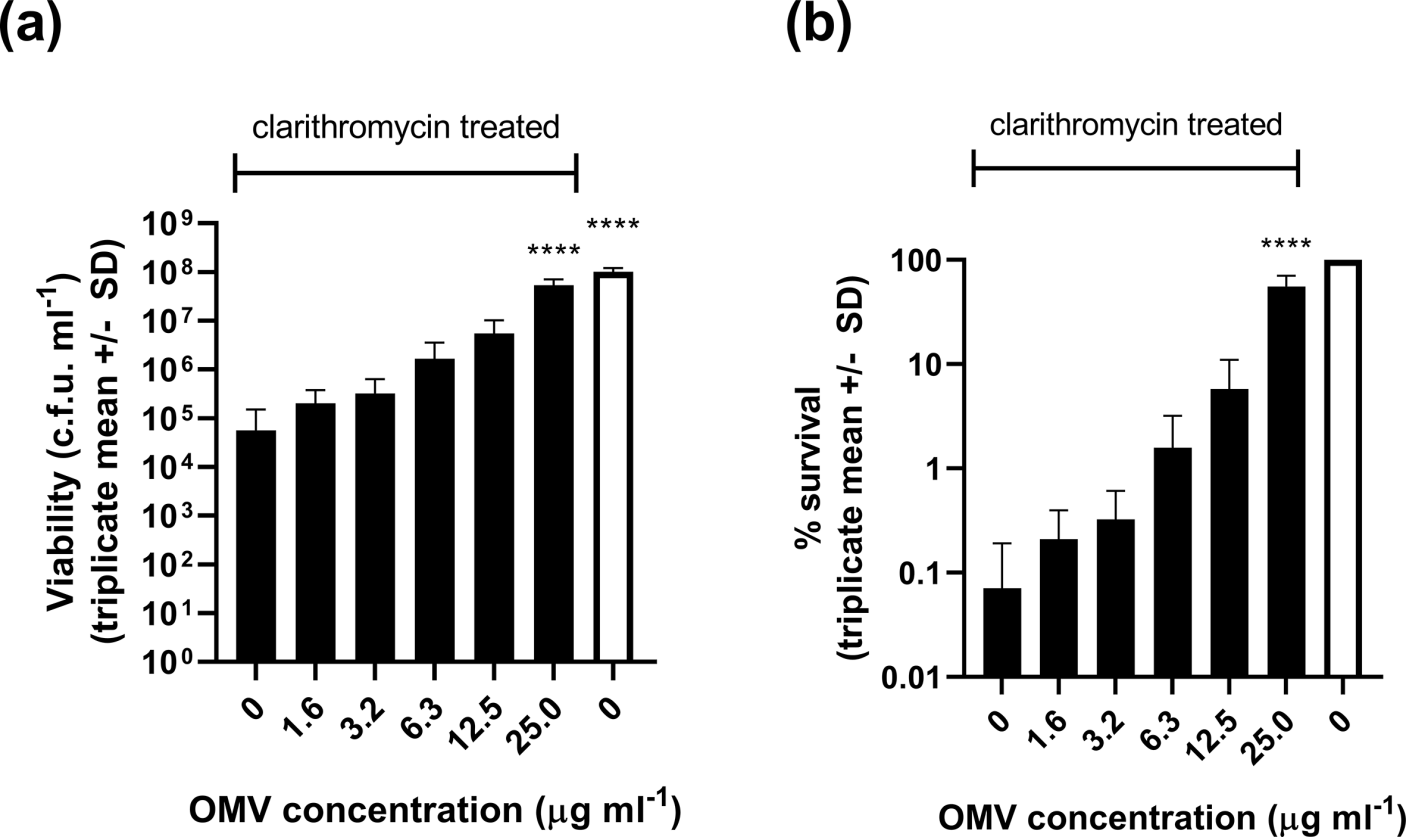
OMV-mediated protection of *H. pylori* against clarithromycin treatment is dose-dependent. Bacteria were suspended in BHI broth and exposed to 5 µg clarithromycin ml^−1^ (black bars) with and without the addition of the indicated concentrations of purified OMVs. After 3 h incubation at 37 °C under microaerobic conditions, the surviving bacteria were quantified by serial dilution and plating. Data are expressed as c.f.u. ml^−1^ (a) and percentage survival compared to untreated bacteria (b). Untreated bacteria (white bars) were incubated in BHI broth without clarithromycin and OMVs. Mean values ± sd from three independent replicates are shown. Addition of 25 µg OMVs ml^−1^ significantly protected *H. pylori* against clarithromycin treatment (****P<0.0001, one-way ANOVA with Dunnett’s multiple comparison test versus the clarithromycin-treated control without addition of OMVs).

**Fig. 5.**
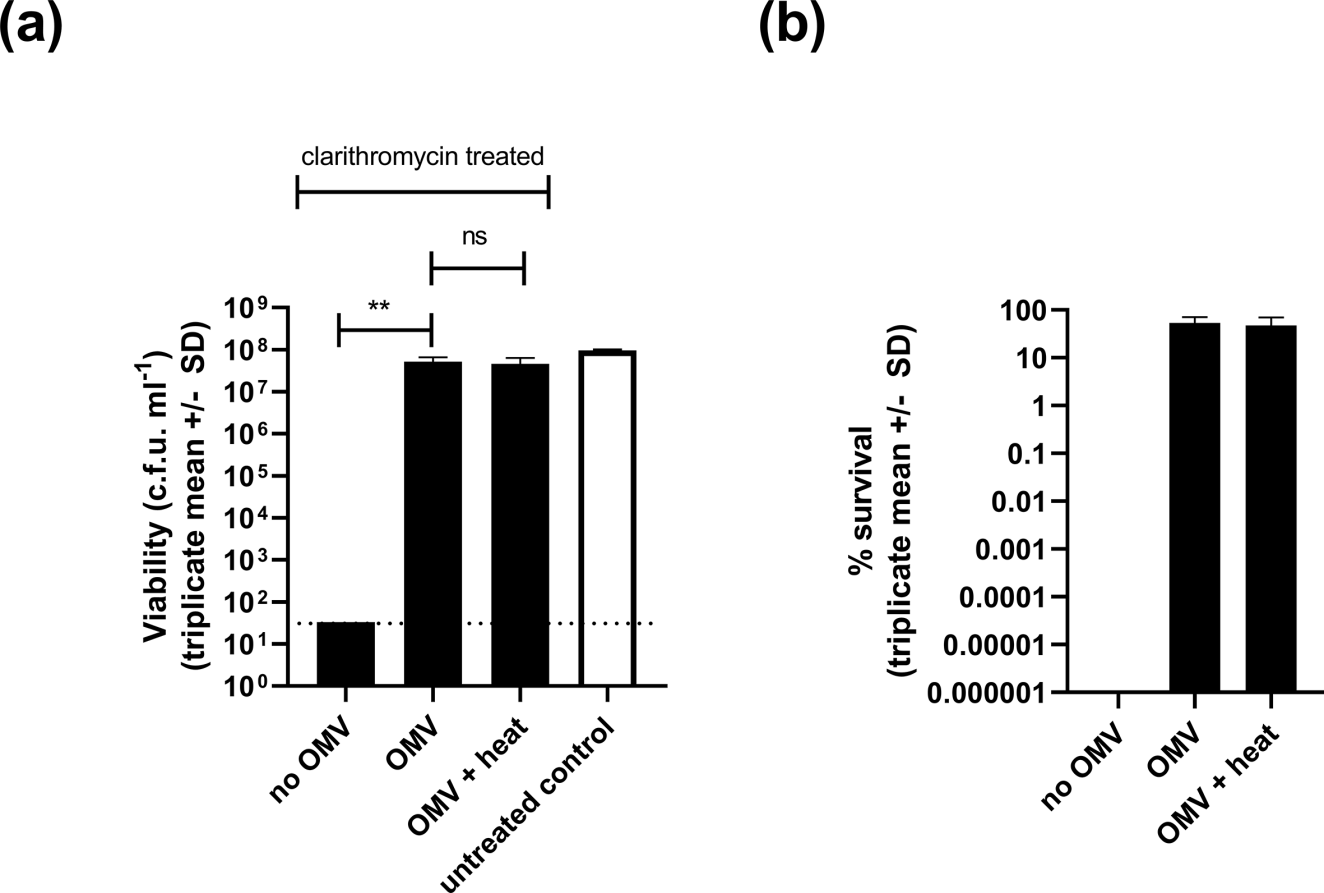
Heat treatment does not prevent OMV-mediated protection of *H. pylori* against clarithromycin. Bacteria were suspended in BHI broth and exposed to 5 µg clarithromycin ml^−1^ (black bars) with and without 25 µg purified OMVs ml^−1^. The OMV label indicates that normal purified OMVs were added. OMV + heat indicates that the OMVs were pre-treated with heat (80 °C for 10 min) before use in the assay. After 3 h incubation at 37 °C under microaerobic conditions, the surviving bacteria were quantified by serial dilution and plating. Data are expressed as c.f.u. ml^−1^ (a) and percentage survival compared to untreated bacteria (b). Untreated bacteria (white bar) were incubated in BHI broth without clarithromycin and OMVs. Mean values ± sd from three independent replicates are shown. The limit of detection, 33 c.f.u. ml^−1^, is indicated by a dashed line. Addition of 25 µg OMVs ml^−1^ significantly protected *H. pylori* against clarithromycin treatment (**P<0.01, one-way ANOVA with Tukey’s multiple comparison tests) and there was no significant difference in the protective effects of heat-treated versus non-heat-treated OMVs. ns, No significant difference.

Since the protective effect of OMVs was most dramatic for clarithromycin treatment, we studied it in more detail. We reduced the clarithromycin concentration from 10 to 5 µg ml^−1^ (sufficient to cause a several log reduction in bacterial viability) and tested a range of OMV concentrations for potential protective effects. OMVs protected *H. pylori* against clarithromycin-mediated killing in a dose-dependent manner (Fig. 4) and the protective effect of OMVs was not ablated by pre-heating them to 80 °C for 10 min to inactivate any OMV-associated enzymes (Fig. 5).

## Discussion

Constitutive production of OMVs during growth is now understood to be a well-characterized and highly conserved feature of all Gram-negative bacteria studied to date. Continuous packaging and export of cellular components is energetically expensive, so OMV secretion must perform some important beneficial functions. Given that OMV production is upregulated in response to stress in some species, the contribution of vesiculation to bacterial survival of environmental stress has been proposed as one such universal benefit of OMV production [17–19].

*H. pylori* is able to persist in the harsh environment of the human stomach for decades, despite a vigorous immune response by the host [1]. Consistent with the recent report by Lekmeechai *et al*. [23], but using a different *H. pylori* strain, we found that addition of purified OMVs could enhance *H. pylori* survival of hydrogen peroxide treatment in a dose-dependent manner. Using a *katA* mutant, Lekmeechai *et al.* [23] showed that the protective effects of *H. pylori* OMV against hydrogen peroxide were mediated by the catalase enzyme.

We also found that OMVs were protective against the antimicrobial peptide LL-37 that is produced by human gastric epithelial cells in response to infection and is bactericidal to *H. pylori* [24]. This protective effect is presumably due to sequestration of LL-37 by OMVs, as previously shown for other membrane active antimicrobial peptides in other bacterial species, for example *E. coli* and *P. syringae* OMVs versus colistin and melittin [20, 22], and *V. cholerae* versus polymyxin B and LL-37 [26].

Addition of OMVs alleviated the LL-37-mediated inhibition of *H. pylori* growth at all LL-37 doses tested but, curiously, growth of OMV-supplemented *H. pylori* was greatest at the highest concentrations of LL-37. It is unclear how OMV addition might have caused this observed trend, but it is possible that the lysing of OMVs by LL-37 resulted in the dispersal of packaged nutrients for the surviving *H. pylori* cells. Alternatively, proteolytic enzymes in the OMVs might have digested LL-37, effectively supplementing the culture media with amino acids to promote bacterial growth. Further mechanistic studies will be needed to characterize the interactions between bacterial OMVs and LL-37.

Treatment of *H. pylori* and other bacterial infections is becoming increasingly difficult due to the development of antibiotic resistance. Improved understanding of bacterial mechanisms of antibiotic resistance and tolerance could help inform the design of new treatments, so we investigated the potential for OMVs to protect *H. pylori* against exposure to antibiotics. Although OMV-mediated protection against β-lactam antibiotics has been reported for other species, for example *Staphylococcus aureus* [27] and *Acinetobacter baumannii* [28], this protection was dependent on the activity of β-lactamase enzyme exported with or inside the vesicles. Since the development of amoxicillin resistance in *H. pylori* does not depend on β-lactamase production [29], it is unsurprising that *H. pylori-*derived OMVs were not directly protective against amoxicillin.

We observed a modest protective effect of OMVs against levofloxacin treatment, but not against metronidazole. In contrast, OMV-mediated protection of *H. pylori* against exposure to clarithromycin was dramatic and dose-dependent. OMVs were still able to protect *H. pylori* against clarithromycin treatment after heat treatment at 80 °C, indicating that a heat-labile enzymatic activity was not likely to be responsible for this protective effect, although more comprehensive analysis will be required to definitely rule out an enzyme-based mechanism. Clarithromycin is a macrolide antibiotic that inhibits protein synthesis by targeting the 23S rRNA region in the 50S ribosomal subunit. Mutations A2143G and A2142G/C in the target region of the 23S rRNA are the most common causes of clarithromycin resistance in *H. pylori* in Europe (reviewed by Xuan *et al*. [30]). The mechanism driving OMV-mediated protection of *H. pylori* against clarithromycin in our study has not yet been elucidated. Simple sequestration is one possible explanation – clarithromycin is a hydrophobic antibiotic and enters bacterial cells via lipid-mediated passive diffusion [31, 32], and macrolide antibiotics can bind directly to lipid membranes [33], so OMVs may have acted as a decoy taking up clarithromycin that would otherwise have diffused into bacterial cells. However, levofloxacin has intermediate lipophilicity and metronidazole is also lipophilic, so a comprehensive study of drug–OMV interactions is needed to assess the potential for vesicles to adsorb each drug. The presence or absence of potential molecular targets for clarithromycin in OMVs, and their binding affinities for the drug, should also be determined. Recent genomic analysis of clarithromycin-sensitive and -resistant *H. pylori* strains has identified additional mutations associated with clarithromycin susceptibility, including membrane proteins [34], and the expression of some outer-membrane proteins is upregulated in clarithromycin-resistant strains [35].

Further work is needed to elucidate the mechanisms by which *H. pylori* OMVs can protect the bacteria against LL-37 and clarithromycin, and to assess whether OMV-mediated survival and growth promotion are biologically relevant *in vivo*. It is not yet clear what concentrations of OMVs are present *in vivo* during bacterial infections, but it is possible that local OMV concentrations could become relatively high in the context of biofilm and/or thick mucus layers. Further study of *in vivo* OMV production is needed.

In some other bacterial species, exposure to stressors has been shown to cause upregulation of vesiculation [19] and/or modulation of OMV contents [26], and it would be useful to determine which antimicrobial treatments might modulate the rate of vesiculation by *H. pylori *. Comprehensive mapping of the OMV biogenesis pathways of *H. pylori* is also needed, to identify candidate targets for novel anti-vesiculation drugs. If such drugs could be developed, they might reduce bacterial stress survival and virulence by disabling OMV production, in turn potentiating the antibacterial activities of conventional antibiotics and the host immune response.

## References

[1] Peek RM, Fiske C, Wilson KT (2010). Role of innate immunity in *Helicobacter pylori*-induced gastric malignancy. Physiol Rev.

[2] Atherton JC, Blaser MJ (2009). Coadaptation of *Helicobacter pylori* and humans: ancient history, modern implications. J Clin Invest.

[3] Atherton JC (2006). The pathogenesis of *Helicobacter pylori*-induced gastro-duodenal diseases. Annu Rev Pathol.

[4] Thung I, Aramin H, Vavinskaya V, Gupta S, Park JY (2016). Review article: the global emergence of *Helicobacter pylori* antibiotic resistance. Aliment Pharmacol Ther.

[5] Malfertheiner P, Megraud F, O'Morain CA, Gisbert JP, Kuipers EJ (2017). Management of *Helicobacter pylori* infection-the Maastricht V/Florence consensus report. Gut.

[6] Savoldi A, Carrara E, Graham DY, Conti M, Tacconelli E (2018). Prevalence of antibiotic resistance in *Helicobacter pylori*: a systematic review and meta-analysis in World Health Organization regions. Gastroenterology.

[7] Miendje Deyi VY, Lare MS, Burette A, Ntounda R, Elkilic O (2019). Update of primary *Helicobacter pylori* resistance to antimicrobials in Brussels, Belgium. Diagn Microbiol Infect Dis.

[8] Tacconelli E, Carrara E, Savoldi A, Harbarth S, Mendelson M (2018). Discovery, research, and development of new antibiotics: the WHO priority list of antibiotic-resistant bacteria and tuberculosis. Lancet Infect Dis.

[9] Keenan J, Day T, Neal S, Cook B, Perez-Perez G (2000). A role for the bacterial outer membrane in the pathogenesis of *Helicobacter pylori* infection. FEMS Microbiol Lett.

[10] Fiocca R, Necchi V, Sommi P, Ricci V, Telford J (1999). Release of *Helicobacter pylori* vacuolating cytotoxin by both a specific secretion pathway and budding of outer membrane vesicles. Uptake of released toxin and vesicles by gastric epithelium. J Pathol.

[11] Schwechheimer C, Kuehn MJ (2015). Outer-membrane vesicles from Gram-negative bacteria: biogenesis and functions. Nat Rev Microbiol.

[12] Mullaney E, Brown PA, Smith SM, Botting CH, Yamaoka YY (2009). Proteomic and functional characterization of the outer membrane vesicles from the gastric pathogen *Helicobacter pylori*. Proteomics Clin Appl.

[13] Olofsson A, Vallström A, Petzold K, Tegtmeyer N, Schleucher J (2010). Biochemical and functional characterization of *Helicobacter pylori* vesicles. Mol Microbiol.

[14] Olofsson A, Nygård Skalman L, Obi I, Lundmark R, Arnqvist A (2014). Uptake of *Helicobacter pylori* vesicles is facilitated by clathrin-dependent and clathrin-independent endocytic pathways. mBio.

[15] Turner L, Bitto NJ, Steer DL, Lo C, D'Costa K (2018). *Helicobacter pylori* outer membrane vesicle size determines their mechanisms of host cell entry and protein content. Front Immunol.

[16] MacDonald IA, Kuehn MJ (2012). Offense and defense: microbial membrane vesicles play both ways. Res Microbiol.

[17] MacDonald IA, Kuehn MJ (2013). Stress-induced outer membrane vesicle production by *Pseudomonas aeruginosa*. J Bacteriol.

[18] Schwechheimer C, Kuehn MJ (2013). Synthetic effect between envelope stress and lack of outer membrane vesicle production in *Escherichia coli*. J Bacteriol.

[19] McBroom AJ, Kuehn MJ (2007). Release of outer membrane vesicles by Gram‐negative bacteria is a novel envelope stress response. Mol Microbiol.

[20] Kulkarni HM, Nagaraj R, Jagannadham MV (2015). Protective role of *E. coli* outer membrane vesicles against antibiotics. Microbiol Res.

[21] Manning AJ, Kuehn MJ (2011). Contribution of bacterial outer membrane vesicles to innate bacterial defense. BMC Microbiol.

[22] Kulkarni HM, Swamy CVB, Jagannadham MV (2014). Molecular characterization and functional analysis of outer membrane vesicles from the Antarctic bacterium *Pseudomonas syringae* suggest a possible response to environmental conditions. J Proteome Res.

[23] Lekmeechai S, Su Y-C, Brant M, Alvarado-Kristensson M, Vallström A (2018). *Helicobacter pylori* outer membrane vesicles protect the pathogen from reactive oxygen species of the respiratory burst. Front Microbiol.

[24] Hase K, Murakami M, Iimura M, Cole SP, Horibe Y (2003). Expression of LL-37 by human gastric epithelial cells as a potential host defense mechanism against *Helicobacter pylori*. Gastroenterology.

[25] Winter J, Letley D, Rhead J, Atherton J, Robinson K (2014). *Helicobacter pylori* membrane vesicles stimulate innate pro- and anti-inflammatory responses and induce apoptosis in Jurkat T cells. Infect Immun.

[26] Duperthuy M, Sjöström AE, Sabharwal D, Damghani F, Uhlin BE (2013). Role of the *Vibrio cholerae* matrix protein BAP1 in cross-resistance to antimicrobial peptides. PLoS Pathog.

[27] Lee J, Lee E-Y, Kim S-H, Kim D-K, Park K-S (2013). *Staphylococcus aureus* extracellular vesicles carry biologically active β-lactamase. Antimicrob Agents Chemother.

[28] Liao Y-T, Kuo S-C, Chiang M-H, Lee Y-T, Sung W-C (2015). *Acinetobacter baumannii* extracellular OXA-58 is primarily and selectively released via outer membrane vesicles after Sec-dependent periplasmic translocation. Antimicrob Agents Chemother.

[29] Co E-MA, Schiller NL (2006). Resistance mechanisms in an in vitro-selected amoxicillin-resistant strain of *Helicobacter pylori*. Antimicrob Agents Chemother.

[30] Xuan S-H, Wu L-P, Zhou Y-G, Xiao M-B (2016). Detection of clarithromycin-resistant *Helicobacter pylori* in clinical specimens by molecular methods: a review. J Glob Antimicrob Resist.

[31] Doucet-Populaire F, Capobianco JO, Zakula D, Jarlier V, Goldman RC (1998). Molecular basis of clarithromycin activity against *Mycobacterium avium* and *Mycobacterium smegmatis*. J Antimicrob Chemother.

[32] Delcour AH (2009). Outer membrane permeability and antibiotic resistance. Biochim Biophys Acta.

[33] Kosol S, Schrank E, Krajačić MB, Wagner GE, Meyer NH (2012). Probing the interactions of macrolide antibiotics with membrane-mimetics by NMR spectroscopy. J Med Chem.

[34] Chen J, Ye L, Jin L, Xu X, Xu P (2018). Application of next-generation sequencing to characterize novel mutations in clarithromycin-susceptible *Helicobacter pylori* strains with A2143G of 23S rRNA gene. Ann Clin Microbiol Antimicrob.

[35] Smiley R, Bailey J, Sethuraman M, Posecion N, Showkat Ali M (2013). Comparative proteomics analysis of sarcosine insoluble outer membrane proteins from clarithromycin resistant and sensitive strains of *Helicobacter pylori*. J Microbiol.

